# Transcriptional repression during spermatogenesis in *C. elegans* requires TOP-2, condensin II, and the MET-2 H3K9 methyltransferase.

**DOI:** 10.17912/micropub.biology.000933

**Published:** 2023-08-23

**Authors:** Emilie Chien, W. Matthew Michael

**Affiliations:** 1 Molecular and Computational Biology Section, Department of Biological Sciences, University of Southern California, Los Angeles, California, United States

## Abstract

In *C. elegans* RNA polymerase II (RNAPII) transcription is globally repressed as oocytes prepare for meiosis. Recent work has identified the transcriptional repressors responsible for genome silencing in oocytes, and they are topoisomerase II ( TOP-2 ), condensin II, the H3K9 methyltransferase SET-25 and MET-2 , and the PIE-1 protein. Here, we focus on TOP-2 , condensin II, and MET-2 and ask if they play a similar role during spermatogenesis. We report that spermatocytes undergo transcriptional repression, as inferred by a deactivation of RNAPII, and this requires TOP-2 , the CAPG-2 subunit of condensin II, and the histone methyltransferase MET-2 .

**Figure 1. Diplotene/diakinesis spermatocytes treated with top-2 , capg-2 , and met-2 RNAi exhibit increased RNAPIIpSer2 signals.**
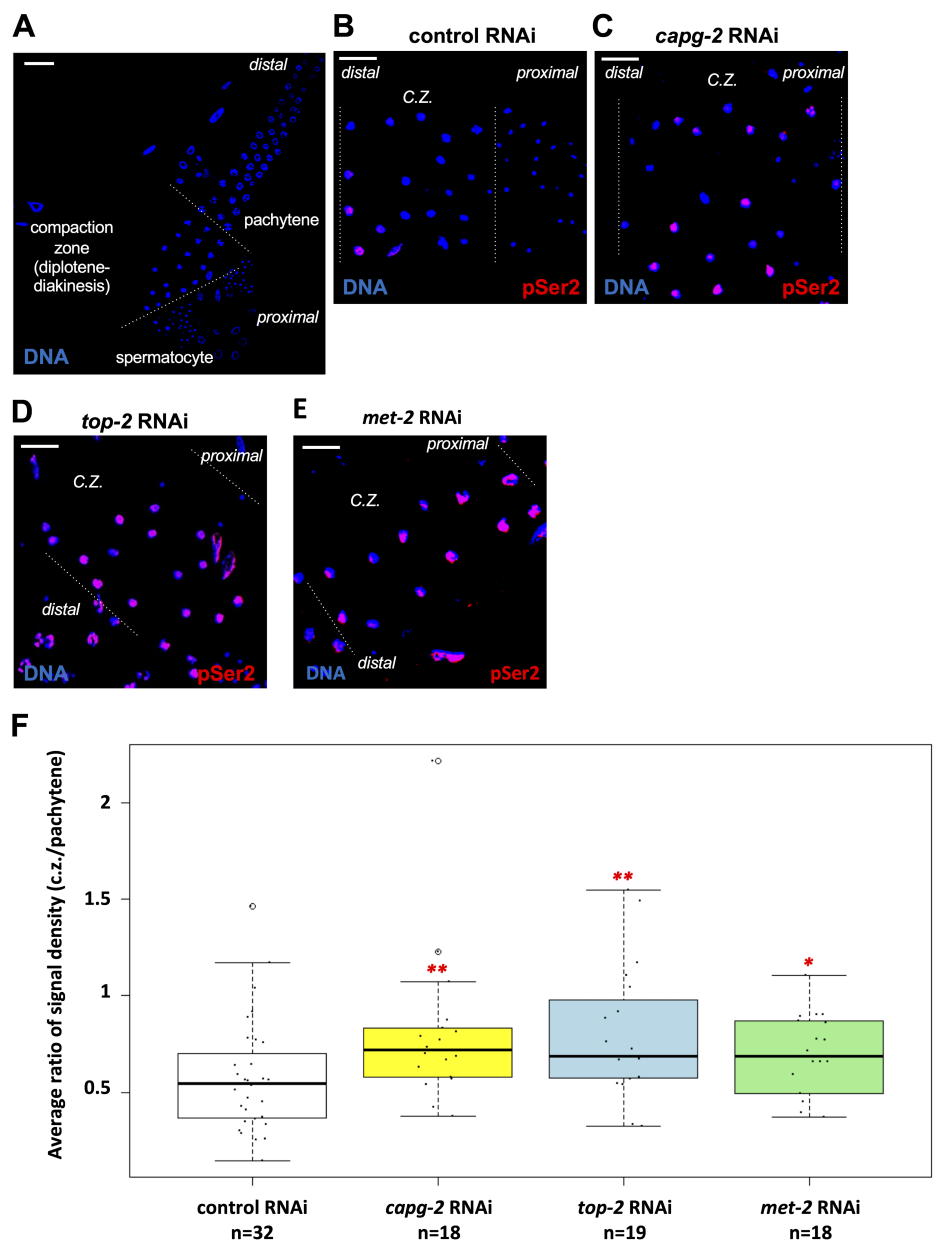
**A.** Dissected wild-type male gonads were fixed and stained for DNA (blue). The gonad is divided into three regions based on DNA morphology. The compaction zone, comprised of diplotene and diakinesis spermatocytes, was examined in subsequent experiments. Scale bar equals 10 μM. **B.** Gonads from male animals treated with control RNAi were fixed and stained for RNAPII (pSer2; red) and DNA (blue). Representative image of the compaction zone (C.Z.) is shown. Scale bar equals 10 μM. **C.** Gonads from male animals treated with *capg-2* RNAi were fixed and stained for RNAPII (pSer2; red) and DNA (blue). Representative image of the compaction zone (C.Z.) is shown. Scale bar equals 10 μM. **D.** Gonads from male animals treated with *top-2* RNAi were fixed and stained for RNAPII (pSer2; red) and DNA (blue). Representative image of the compaction zone (C.Z.) is shown. Scale bar equals 10 μM. **E.** Gonads from male animals treated with *met-2* RNAi were fixed and stained for RNAPII (pSer2; red) and DNA (blue). Representative image of the compaction zone (C.Z.) is shown. Scale bar equals 10 μM. **F.** Quantification of data presented in panels B-E. RNAPIIpSer2 signal was measured using ImageJ. The average ratio of normalized raw integrated density is plotted on the y-axis, and this corresponds to the ratio of signal intensities derived from the compaction zone (C.Z.) over the pachytene zone. Samples were analyzed for each RNAi treatment over at least 2 independent replicates. Significance was measured using Wilcoxon Rank Sum test. The number of samples analyzed for each condition is shown below. **p<0.01; *p<0.05.

## Description

In many organisms, including nematodes, fruit flies, and mice, transcription is globally repressed during gametogenesis (Moore and Lintern-Moore, 1974; Schisa et al., 2001; Walker et al., 2007; Navarro-Costa et al., 2016; Abe et al., 2018; Belew et al., 2023) . In *C. elegans,* oocytes globally repress RNAPII-mediated transcription during diakinesis as they prepare for the meiotic divisions (Walker et al., 2007; Belew et al., 2023) , and they do so using an array of genome silencing factors such as the TOP-2 /condensin II axis, the H3K9me/heterochromatin pathway, and the PIE-1 protein. The TOP-2 /condensin II axis, which is best known for chromosome condensation in mitotic cells (Lau and Csankovszki, 2014; Kagami and Yoshida, 2016; Batty and Gerlich, 2019) , is also required for germline genome silencing when L1 larvae undergo starvation (Belew et al., 2021) . In this work we asked two questions. One, do developing spermatocytes undergo a genome silencing event in *C. elegans* ? And two, if so, does this require TOP-2 , condensin II, and/or MET-2 ?

To get the answers, we performed immunofluorescent staining of dissected male gonads with an antibody that detects a phospho-epitope at the second serine on the carboxy-terminal domain of RNAPII (referred to as pSer2 for the remainder of this study). Active and elongating RNAPII contains the pSer2 mark (Palancade and Bensaude, 2003; Abuhashem et al., 2022) , and as such pSer2 has been used as a marker for nuclei that are actively transcribing mRNAs (Palancade and Bensaude, 2003) . In *C. elegans* pSer2 staining has been used widely to study transcriptional control on a global level (Seydoux and Dunn, 1997; Walker et al., 2007; Guven-Ozkan et al., 2008; Shakes et al., 2009; Bowman et al., 2013; Belew et al., 2021; Belew et al., 2023) . We started by dividing the male gonad into three sections based on DNA morphology ( Figure 1A ). The first group, the pachytene region, contains cells that have the least compacted chromatin, as reflected by a Feret diameter for pachytene nuclei of 4.23+/-0.28 microns (n=49). Second, the diplotene/diakinesis region (also referred to as the compaction zone; Shakes et al., 2009), contains cells with more compact chromatin (Feret diameter of 3.13+/-0.5 microns, n=56). And, finally, spermatids have the most compacted chromatin in the male gonads (Feret diameter of 1.49+/-0.26, n=349). In wild-type male gonads, we saw that almost all the nuclei in the pachytene region had a strong pSer2 signal. The average pSer2 signal decreased when we examined the compaction zone (Figure 2B; quantified in Figure 2F). We next asked if loss of genome silencing factors would perturb the pattern of pSer2 signal observed for the control RNAi samples. The compaction zones of gonads from males treated with *top-2* , *capg-2 ,* or *met-2* RNAi showed an increased number of pSer2-positive nuclei, from 18% with a strong signal in the control to 31%, 32%, and 28% for *top-2 , capg-2 ,* and *met-2* RNAi, respectively. To quantify this experiment, we measured pSer2 signal intensity for all compaction zone nuclei under the four different conditions, and these data are shown in Figure 1F . Depletion of all three genome silencers resulted in a significant increase in pSer2 signals. These results show that the TOP-2 /condensin II mediated transcriptional repression, as well as repression mediated by the H3K9me pathway, occurs in diplotene/diakinesis stage spermatocytes, as it does in oocytes. Therefore, we conclude that this mechanism of transcriptional repression is common to both male and female gametogenesis in *C. elegans.*

## Methods

**Animal growth conditions**

Worms were maintained on 60-mm plates containing nematode growth media (NGM) seeded with the *E. coli* strain OP50 or HT115 . Worms were grown at 20 ^o^ C and propagated through egg preparation (bleaching) to produce a synchronized population. Male DUP75 *C. elegans* were generated by picking 20 L4 hermaphrodites and heat-shocking them at 30 ^o^ C for 5 hours. From their progeny, 8 male and 2 hermaphrodite worms were picked onto new plates to mate. This yielded an F2 population with ~50% male animals. OP50 bacteria served as the primary food source. It was grown in LB media containing 100 µg/ml streptomycin by shaking at 37 ^o^ C overnight. 500 µl of the culture was seeded on Petri dishes containing NGM + streptomycin. HT115 bacteria grown in LB media containing 100 µg/ml carbenicillin and 12.5 µg/ml tetracycline and seeded on NGM + carbenicillin + tetracycline plates were also used as a source of food. Our RNAi strains were obtained from the Ahringer library and verified by Sanger sequencing. Bacteria expressing dsRNA were streaked on LB-agar plates containing 100 µg/ml carbenicillin and 12.5 µg/ml tetracycline and incubated at 37 ^o^ C overnight. Single colonies were picked and grown in 25 ml LB cultures with 100 µg/ml carbenicillin and 12.5 µg/ml tetracycline. 500 µl of this culture was seeded on 60-mm Petri dishes containing 5 mM IPTG.

**RNAi treatment**

RNAi-containing NGM plates were prepared as described in the “ **Animal growth conditions** ” section. HT115 cells transformed with an empty pL4440 vector was used as a negative control. RNAi conditions used in this study and tests for their efficacy is described below:

*top-2* RNAi

8 male adult and 2 hermaphrodite L4 worms were picked onto HT115 food plates to mate for 48 hours. F1 progeny were then moved plated to plates seeded with *top-2* RNAi for another 48 hours. Embryonic lethality was observed at >90%.

*capg-2* RNAi

8 male adult and 2 hermaphrodite L4 worms were picked onto HT115 food plates to mate for 48 hours. F1 progeny were then moved to plates containing *capg-2* RNAi for the remaining 48 hours. An embryonic lethality of 80%-100% was seen with this RNAi treatment.

*met-2* RNAi

8 male adult and 2 hermaphrodite L4 worms were picked onto plates containing *met-2* RNAi for the entirety of their life cycle. To test for RNAi efficacy, WMM1 L1 worms were grown on *met-2* RNAi plates. Adult worms were bleached and an L1 chromatin compaction assay was performed. See Belew et al., 2021, for details on the L1 compaction assay.

**Antibodies and dilutions**

RNAPII pSer2 : Rabbit antibody from Abcam (ab5095, Cambridge, Massachusetts) was used at 1:100. Secondary antibodies : Alexa Fluor conjugated secondary antibodies from Invitrogen (Thermofisher Scientific, Waltham, Massachusetts) were used at a dilution of 1:200.

**Immunofluorescence staining**

To obtain gonads, 15 adult male animals were picked into a drop of 15 µl M9 minimal medium on a coverslip. 2 µl of anesthetic (20mM Sodium Azide and 0.8M Tetramisole hydrochloride) was added to immobilize them. Animals were dissected using 25Gx5/8 needles (Sigma Aldrich, St. Louis, Missouri). To release gonads, adult animals were cut twice, once right below their pharyngeal bulb and once near the tail. The coverslip was then mounted onto a poly-L-lysine covered slide and excess liquid was removed via capillary action. Slides were put on dry ice for 30 minutes. Samples were then freeze-cracked by flicking the coverslips off for permeabilization. Once permeabilized, slides were put in ice-cold 100% methanol for 2 minutes and then fixing solution (0.08M HEPES pH 6.9, 1.6mM MgSO4, 0.8mM EGTA, 3.7% formaldehyde, 1X phosphate-buffered saline) for another 30 minutes. After fixing, slides were washed three times with TBS-T (TBS with 0.1% Tween-20) and were blocked for 30 minutes with TNB (containing 100mM Tris-HCl, 200 mM NaCl, and 1% BSA). Primary antibodies were then applied at the dilutions described above in TNB and slides were incubated at 4 ^o^ C overnight. On day 2, primary antibodies were washed 3 times with TBS and slides were incubated with secondary antibodies and Hoechst 33342 dye for 2 hours at room temperature. Slides were washed 3 times with TBS, then mounting medium (50% glycerol in PBS) and coverslips were applied and sealed with Cytoseal XYL (Thermofisher).

**Immunofluorescent imaging**

All slides were imaged using an Olympus Fluoview FV1000 confocal microscope using Fluoview Viewer software at a magnification of 600x (60x objective and 10x eyepiece magnification). Laser intensity was controlled for experiments to achieve consistency among samples. Images were analyzed using ImageJ software.

**Quantification of data**

Nuclear diameter measurements

Images were analyzed using ImageJ. In brief, outlines of Hoechst-stained nuclei in the different gonadal regions were created and the particles were analyzed to obtain a Feret’s diameter measurement in µm for each nucleus. Data were collected from 5 distinct gonad samples.

RNAPIIpSer2 signal quantification

Images were analyzed using ImageJ. An outline of all Hoechst-stained nuclei in the compaction zone was created to mark the space occupied by DNA. The region of interest was copied and pasted to the RNAPIIpSer2 signal image and the raw integrated density (the sum of the values of the pixels in the selection) was measured. The raw integrated density was then normalized by the area to get a measure we called “signal density”. To account for possible variability in signal intensity due to different degrees of antibody penetration from sample to sample, each signal density was normalized to an average signal density from 5 pachytene nuclei found in the same image. The final normalized signal densities for the compaction zone are presented. For each condition, data were collected from at least two independently performed experimental replicates and the data were then pooled for statistical analysis and presentation.

**Statistical analysis**

Prior to performing any statistical test, data was tested whether it was parametric or not. To do so, the Shapiro-Wilk test was used to test for normal distribution and F-test was used to test for variance homogeneity of the datasets we were comparing. Data were then analyzed using a Student’s t-test or Wilcoxon Rank Sum test depending on whether the datasets fulfill the requirements for a parametric test or not. Differences between any two datasets were considered statistically significant if a P-value of <0.05 was obtained.

## Reagents

**Table:** 

STRAIN	GENOTYPE	AVAILABLE FROM
DUP75	*pgl-1 ( sam33 [ pgl-1 ::GFP::3xFLAG]) IV *	*Upon request*
WMM1	*bnIs1 [ pie-1 ::gfp:: pgl-1 + unc-119 (+)]; ltls37 [(pAA64) pie-1 p::mCherry:: his-58 + unc-119 (+)] IV. *	*Upon request*
